# Phase‐inversion constructed Mo_2_C@NC microreactor with optimized pyridinic N p‐band center for high‐performance Li–S batteries

**DOI:** 10.1002/smo2.70051

**Published:** 2026-05-06

**Authors:** Fangyi Chu, Helong Jiang, Xiangcun Li, Xuri Wang, Gaohong He, Xiaobin Jiang, Xuehua Ruan

**Affiliations:** ^1^ State Key Laboratory of Fine Chemicals Department of Chemical Engineering Dalian University of Technology Dalian China; ^2^ Department of Energy and Chemical Engineering Panjin Campus Dalian University of Technology Panjin China

**Keywords:** batteries, microreactor, nitrogen‐doped carbon, p‐band, phase‐inversion

## Abstract

Nitrogen‐doped carbon (NC) is widely employed as a conductive matrix in Li‐S batteries, yet its intrinsic catalytic contribution is often overlooked when combined with metal compounds. Herein, we propose a hierarchical microreactor architecture that integrates conductive and catalytic functions through an intimately coupled Mo_2_C@NC interface. Density functional theory calculations demonstrate that coupling NC with Mo_2_C induces pronounced electron redistribution of N atoms, with pyridinic N exhibiting the strongest charge transfer from Mo_2_C, making the p‐band center closest to the Fermi level, thereby endowing superior LiPSs adsorption activity. Guided by this insight, a phase‐inversion strategy is employed using pyridinic‐N‐rich polyacrylonitrile (PAN) and MoO_3_ precursors to construct a cross‐linked MoO_3_@PAN network, which is subsequently transformed into Mo_2_C@NC microreactors after carbonization. In this structure, Mo_2_C nanowires are uniformly confined within pyridinic‐N‐rich carbon shells, forming a zero‐distance conductive–catalytic interface that enables efficient electron transfer from Mo_2_C to NC. This integrated microreactor provides continuous electron pathways, abundant catalytic sites, and unobstructed ion transport, effectively avoiding pore blockage commonly encountered in conventional composite cathodes. Consequently, the Mo_2_C@NC cathode exhibits high cycling stability over 1000 cycles at 2.0 C with a low decay rate of 0.052% per cycle. Even at 4.0 C, it retains 790.6 mAh g^−1^ for over 400 cycles with only 0.027% fading per cycle.

## Graphical abstract

A phase‐inversion constructed Mo_2_C@NC microreactor activates the intrinsic catalysis of nitrogen‐doped carbon via interfacial electron redistribution. Strong charge transfer to pyridinic N optimizes the p‐band center, enabling efficient lithium polysulfide adsorption, fast redox kinetics, and long‐term stable, high‐rate Li–S battery performance.

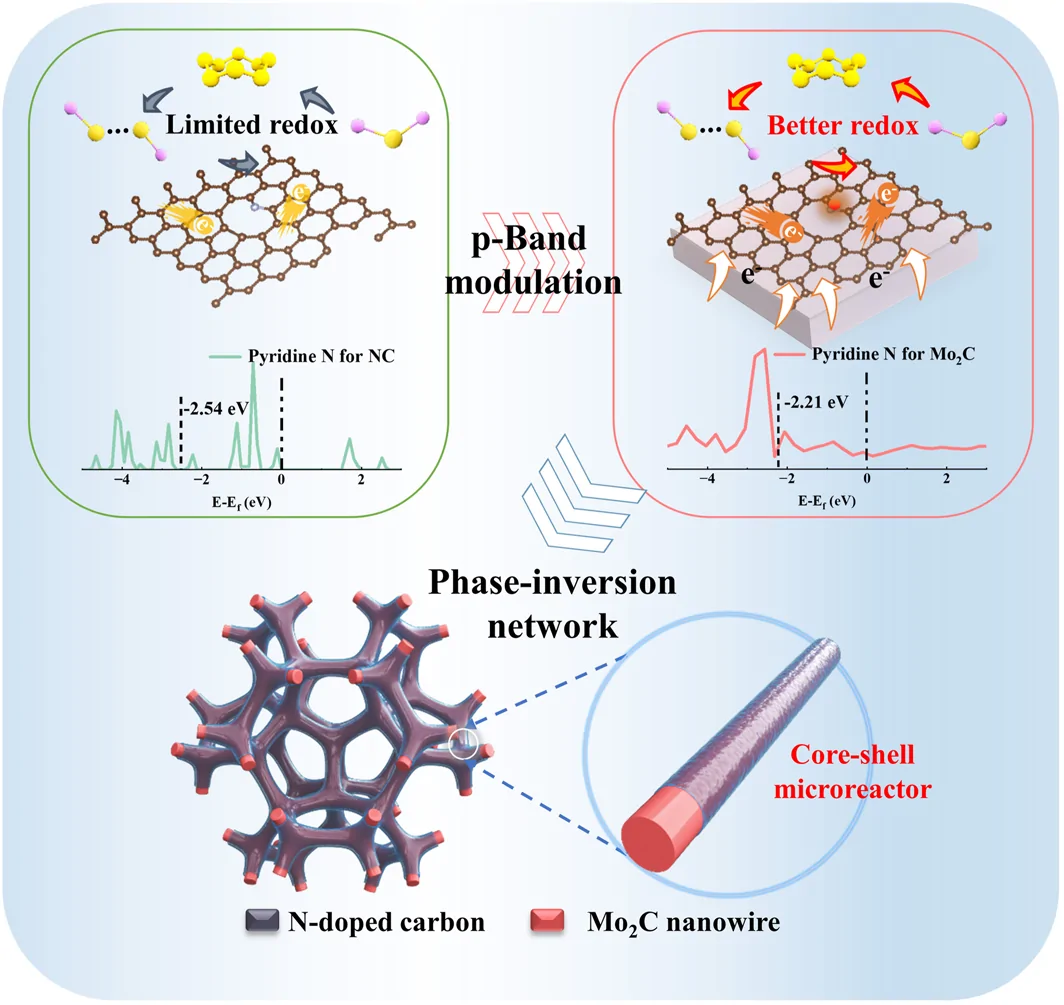

## 1 INTRODUCTION

With the rapid development of renewable energy,^[1]^ lithium‐sulfur (Li‐S) batteries have emerged as an attractive candidate for next‐generation energy storage devices due to their ultrahigh theoretical energy density (2600 Wh kg^−1^) and the abundance and low cost of sulfur.^[2]^ Despite these advantages, the commercial of Li‐S is still impeded by sluggish redox reaction kinetics,^[3]^ poor conductivity of sulfur and the severe shuttle effect of lithium polysulfides^[4]^ (LiPSs), which result in rapid capacity fading and poor rate capability.^[5]^

Carbon‐based materials such as carbon nanotubes[^6^, ^7^, ^8^] and graphene[^7^, ^9^] etc., have been extensively utilized as conductive hosts for sulfur cathodes owing to their excellent electrical conductivity and structural tunability. However, their intrinsic nonpolar nature leads to weak chemical interactions with polar LiPSs, hindering their catalytic efficiency and ability to suppress the shuttle effect. To address this, numerous studies have integrated carbon matrices to enhance the adsorption and conversion of LiPSs. Among these, nitrogen‐doped carbon (NC) has been widely applied as a conductive matrix due to its tunable electronic structure and decent conductivity. However, its intrinsic catalytic efficiency remains limited.^[10]^

Previous studies have shown that coupling N‐doped carbon with metal compounds can induce electronic redistribution, thereby boosting interfacial reactivity and enabling the construction of more effective microreactors.[^11^, ^12^] In the integration of nitrogen doping with metal compounds, architectures of various dimensions can be rationally designed. According to the previous reports,^[9]^ due to the intrinsic lattice orientation limitations of polar materials, their common morphologies are typically lamellar,^[13]^ spherical,^[14]^ or one‐dimensional (1D) linear structures.^[15]^ 1D nanowires, with their high aspect ratio and continuous morphology, provide long‐range ion transport pathways and abundant exposed surfaces, thereby offering advantages for electrochemical kinetics. As to the dimension of N‐doped carbon, diverse structures can be adopted depending on the selected template or suitable precursors,^[16]^ thereby ensuring strong interfacial coupling between carbon and polar materials. In such designs, the polar material acts as an adsorption‐active site, while the porous carbon matrix facilitates lithium‐ion conduction within the electrolyte.^[17]^

In this regard, if NC is considered as the catalytic reaction center and nicely coupled with metal compounds based on the morphology of metal compounds, NC might also exhibit enhanced activity. This enables the construction of a conductive framework that combines both electrical conductivity and catalytic activity, achieving zero distance between the two and markedly improving mass transfer efficiency while accelerating the overall catalytic reaction process. Based on this concept, a combined model of NC and polar materials can be established. Density functional theory (DFT) calculations can afterward be used to confirm that the catalytic activity of NC is indeed modulated by coupling with polar materials and such interaction is favorable for polysulfide adsorption. This theoretical verification can be further validated through rational experimental design.^[14]^

After confirming the DFT simulation results, the coating structure can be further optimized. Specifically, we propose the design of a microreactor in which 1D materials act as the framework while NC serves as the coating layer. To ensure sufficient electrolyte penetration and ion transport, the overall structure can be engineered into a three‐dimensional network, thereby providing ample mass transfer space. This three‐dimensional framework not only offers a continuous conductive network but maximizes ion transport channels for lithium ions. By rationally connecting 1D nanowires, a three‐dimensional network can be realized while retaining the intrinsic advantages of 1D structures.^[18]^

In constructing three‐dimensional network‐like microreactors, a phase‐inversion method offers an effective strategy.^[19]^ It enables cross‐linked polymer coatings on 1D backbones, which after carbonization yield rigid porous frameworks with N‐doped carbon shells that combine polarity with high conductivity.^[20]^ In this way, inorganic fillers can be controllably encapsulated and immobilized within polymer coating. After carbonization, the polymer skeleton evolves into a mechanically stable carbon framework that protects the encapsulated fillers from deformation, thereby achieving structural robustness and enhanced catalytic cooperation within the microreactor. In addition, transition metal carbides,^[21]^ such as molybdenum carbide^[22]^ (Mo_2_C), have attracted significant attention owing to their metallic‐level conductivity and Pt‐like catalytic activity. In Li‐S batteries, Mo_2_C not only serves as an efficient polar catalyst for LiPSs adsorption and conversion but also offers high structural and chemical stability.[^23^, ^24^] However, the nature of electronic coupling between Mo_2_C and N‐doped carbon, and its precise role in interfacial catalysis during redox conversion remains insufficiently understood.

In this work, we propose a microreactor architecture coupled with electronic structure modulation to enhance the catalytic conversion of lithium polysulfides in Li–S batteries. DFT calculations were first performed to systematically investigate the coupling behavior between Mo_2_C and different nitrogen coordination environments, including pyridinic N, pyrrolic N, and graphitic N. The results reveal that pyridinic N coupled with Mo_2_C exhibits a p‐band center closest to the Fermi level, stronger electron‐accepting capability, and higher Li_2_S_4_ binding energy. Moreover, the Mo_2_C–pyridinic N interaction significantly lowers the Li_2_S decomposition barrier to 0.88 eV, indicating accelerated redox conversion kinetics.

Guided by these theoretical insights, a core–shell Mo_2_C@NC microreactor was constructed via a phase‐inversion strategy using PAN‐derived pyridinic‐N‐rich carbon as the shell and Mo_2_C as the skeleton. It should be noted that the “microreactor” in this work refers to a nanoscale confined catalytic domain within the solid cathode material, rather than a conventional microfluidic reactor used in chemical engineering.^[25]^ In this architecture, the intimate Mo_2_C–NC interface forms a confined reaction space that integrates electron transfer, LiPS adsorption, and catalytic conversion. Benefiting from the synergistic effects of the microreactor configuration and electronic coupling, the resultant material demonstrates outstanding rate capability and long‐term cycling stability with 1093.6 mAh g^−1^ at 2.0 C and maintained stable cycling over 1000 cycles, and an ultralow capacity decay rate of only 0.0523% per cycle. Even under high‐rate conditions at 4.0 C, the Mo_2_C@NC electrode delivered an initial capacity of 790.6 mAh g^−1^ and an exceptionally low fading rate of 0.027% per cycle. Furthermore, under a high sulfur loading of 5.57 mg cm^−2^, the cell retained stable cycling for 80 cycles with an areal capacity of 4.87 mAh cm^−2^. These results demonstrate that integrating the microreactor design with electronic structure modulation provides an effective strategy to accelerate sulfur redox kinetics and achieve high‐performance Li–S batteries.

## 2 RESULTS AND DISCUSSIONS

We systematically investigated the coordination environments of pyridinic N, pyrrolic N, and graphitic N (Figure 1a), as well as their schematic diagram with molybdenum carbide (Figure 1b). Among these, pyridinic N was the most favorable site for promoting redox in Li‐S batteries when integrated with Mo_2_C (Figure 1c). Representative structural models of graphitic N, pyrrolic N, and pyridinic N are shown in Figure S1, while Figure S2 depicts the models after integrated with Mo_2_C, denoted as GraNC/Mo_2_C, PrNC/Mo_2_C, and PyNC/Mo_2_C, respectively. After coupling, pronounced charge transfer from Mo_2_C to the N‐doped carbon layers was observed (Figure 1d–f), with charge transfer amounts of 6.56, 7.18, and 7.35 e^−^ for GraNC/Mo_2_C, PrNC/Mo_2_C, and PyNC/Mo_2_C, respectively. Notably, PyNC/Mo_2_C exhibited the largest Bader charge transfer, indicative of stronger interfacial electronic interactions. This enhanced interaction was further reflected using the DFT method. The Li_2_S_4_ binding energy of PyNC after coupling with Mo_2_C (Figure 1g) is the strongest adsorption capability compared to that of GraNC/Mo_2_C and PrNC/Mo_2_C (Figure S3), suggesting more favorable reaction kinetics for sulfur redox conversion.^[26]^

**FIGURE 1:**
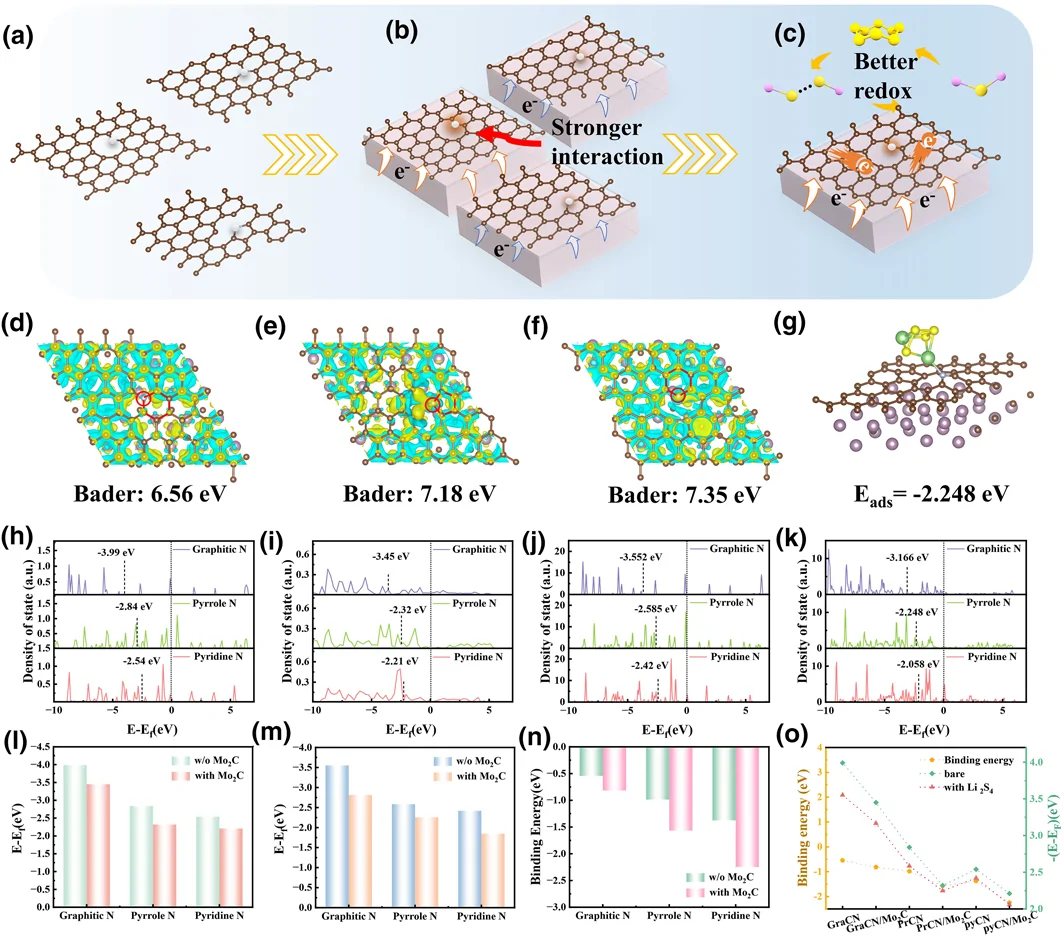
(a) The coordination environments of pyridinic N, pyrrolic N, and graphitic N, (b) corresponding schematic diagrams after coupling with Mo_2_C and (c) redox in Li‐S batteries; structural models of (d) GraNC/Mo_2_C, (e) PrNC/Mo_2_C, and (f) PyNC/Mo_2_C; (g) the Li_2_S_4_ binding energy of PyNC after coupling with Mo_2_C; p‐band centers of N atoms for (h) GraNC, PrNC, and PyNC, (i) GraNC/Mo_2_C, PrNC/Mo_2_C, and PyNC/Mo_2_C, (j) GraNC, PrNC, and PyNC, after Li_2_S_4_ adsorption and (k) GraNC/Mo_2_C PrNC/Mo_2_C, PyNC/Mo_2_C after Li_2_S_4_ adsorption; comparative analysis for the p‐band centers of the six configurations before (l) and after adsorbing Li_2_S_4_ (m); (n) binding energy of the six models (o) statistical correlation analysis between the p‐band center and binding energy.

To further investigate the catalytic mechanisms of NC/Mo_2_C within Li‐S batteries, Density of states (DOS) of GraNC, PrNC, and PyNC before and after coupling with Mo_2_C was given (Figure S4) and we extracted the p‐band centers of N atoms for the three nitrogen configurations (Figure 1h) and their Mo_2_C‐coupled counterparts (Figure 1i) based on the projected DOS analysis. Both PyNC/Mo_2_C exhibited N p‐band centers closest to the Fermi level compared with GraNC/Mo_2_C and PrNC/Mo_2_C, suggesting that Mo_2_C coupling effectively modulates the N p‐orbital energy by shifting it positively toward the Fermi level. After Li_2_S_4_ adsorption, the p‐band centers of N atoms in the six models (GraNC, PrNC, PyNC, and GraNC/Mo_2_C, PrNC/Mo_2_C, PyNC/Mo_2_C) were further analyzed in Figure 1j,k, consistently revealing the superiority of PyNC and PyNC/Mo_2_C. A comparative analysis for the p‐band centers of the six configurations before (Figure 1l) and after adsorbing Li_2_S_4_ (Figure 1m) showed that, regardless of the N coordination environment, coupling with Mo_2_C consistently shifted the p‐band center closer to the Fermi level, with PyNC/Mo_2_C exhibiting the most significant effect. Binding energy of the six models corroborated these findings (Figure 1n), confirming that PyNC/Mo_2_C possessed the strongest interaction with Mo_2_C. Finally, statistical correlation analysis between the p‐band center and binding energy (Figure 1o) revealed a strong positive relationship, reinforcing the validity of the p‐band center as an effective descriptor for polysulfide binding capability.^[27]^ Overall, the introduction of Mo_2_C markedly elevates the p‐band center of nitrogen, enhancing the Li_2_S_4_ adsorption of N‐doped carbon, with pyridinic N showing the strongest electronic interaction with Mo_2_C and the highest binding affinity toward Li_2_S_4_.

Guided by these insights, we designed a composite structure using pyridinic‐N‐rich polyacrylonitrile (PAN) and MoO_3_ as the precursor; after phase‐inversion and carbonization strategy, a cross‐linked MoO_3_@PAN was obtained (Figure S5). Mo_2_C were transformed from MoO_3_ nanowires, and wrapped by pyridinic‐N‐dominated N‐doped carbon layers after carbonization of the as prepared MoO_3_@PAN, named as Mo_2_C@NC (Figure 2a,b). This architecture was employed as the cathode host in Li‐S batteries to validate the optimized catalytic regulation of Mo_2_C on NC and the corresponding electrochemical advantages. The detailed synthesis process is illustrated in Figure S6. MoO_3_ nanowires (Figures 2c and S7) were first synthesized via a hydrothermal method. To achieve a cross‐linked structure, PAN was dissolved in dimethyl formamide (DMF) first and mixed with as prepared MoO_3_ nanowires to form a homogeneous slurry. The mixture was later scraped into a liquid film and immediately transformed into deionized water. In this process, nonsolvent‐induced phase separation occurred, during which DMF rapidly diffused into water leading to the formation of micropores, while the PAN polymer simultaneously coated the surface of MoO_3_ nanowires to construct a continuous network structure. After drying, a membrane with MoO_3_ as core and PAN as the crosslinked coating layer was obtained, denoted as MoO_3_@PAN‐x according to the ratio of MoO_3_ to PAN. Subsequently, the membrane was cut into slices followed by pre‐oxidation and carbonization treatments, which transformed the MoO_3_ nanowires into carbides of manganese (named as Mo_x_C) while converting PAN into a pyridinic‐N‐rich N‐doped carbon coating (named as NC).

**FIGURE 2:**
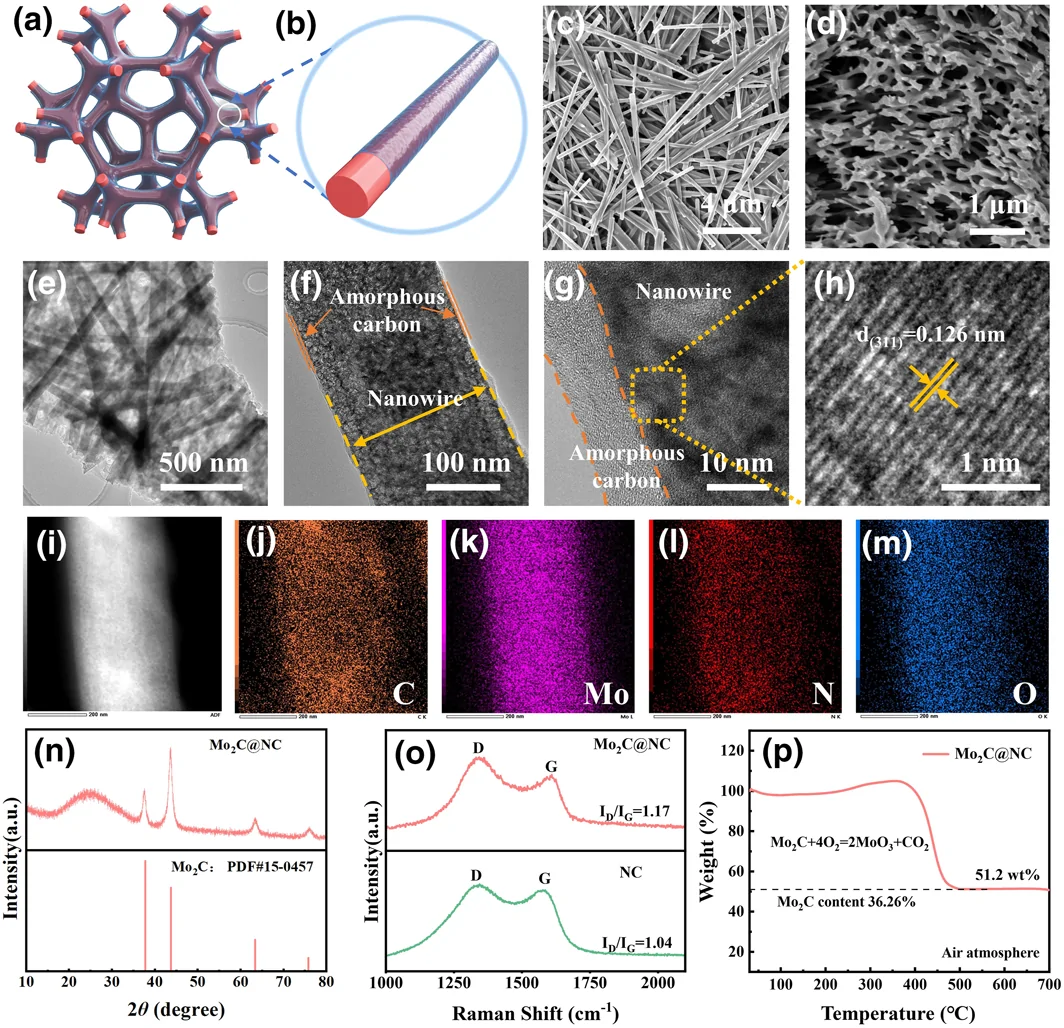
(a, b) Schematic diagram of Mo_2_C@NC; scanning electron microscopy image of (c) MoO_3_ nanowire, and (d) Mo_2_C@NC; (e–h) transmission electron microscopy image of Mo_2_C@NC, (i–m) elemental mapping analysis of Mo_2_C@NC, (n) X‐ray diffraction pattern of Mo_2_C@NC, (o) Raman spectroscopy of Mo_2_C@NC, (p) thermogravimetric analysis of Mo_2_C@NC.

To determine the fundamental reaction conditions governing the conversion of MoO_3_@PAN‐x into Mo_x_C@NC and to optimize the PAN/MoO_3_ ratio, precursor MoO_3_@PAN‐x membranes were fabricated using slurries with different PAN‐to‐MoO_3_ ratios. Six samples were prepared with MoO_3_, PAN, and DMF ratios of 0.75:1:10, 1:1:10, 1.25:1:10, 1.5:1:10, 1.75:1:10, and 2:1:10, respectively. These slurries were cast into membranes under identical conditions, followed by drying, pre‐oxidation, and carbonization to obtain the corresponding samples, denoted as M3, M4, M5, M6, M7, and M8 according to the PAN/MoO_3_ ratio. The crystalline phases of these samples were characterized by X‐Ray diffraction (XRD) (Figure S8). It is worth noting that the ratio of MoO_3_ to PAN plays a critical role in determining the final crystalline phase of the composite. As revealed by XRD analysis, when the MoO_3_: PAN: DMF ratio was adjusted to 1.25:1:10, the obtained product was predominantly Mo_2_C (PDF#15‐0457) with no obvious MoO_2_ (PDF#32‐0671) peaks. In contrast, either an excess or deficiency of MoO_3_ resulted in the appearance of more pronounced MoO_2_ signals. This phenomenon can be ascribed to the delicate balance between Mo and the carbon source during the carbothermal reduction process. Specifically, insufficient carbon derived from PAN cannot completely reduce MoO_3_ to Mo_2_C, leaving residual MoO_2_. Conversely, excessive carbon leads to over‐coating of Mo‐based nanowire, which might hinder the intimate contact between nanowire and NC, thereby preventing full conversion from MoO_3_ to Mo_2_C. We also carried out preliminary cycling tests on Li‐S batteries assembled with these samples (Figure S9). The results revealed that the material with a pure Mo_2_C phase, obtained at a precursor ratio of 1.25:1:10, exhibited the best cycling performance. Therefore, it can be concluded that the optimized MoO_3_ to PAN ratio ensures enough carbon availability for reduction while maintaining efficient Mo‐C interactions, favoring the formation of Mo_2_C coupled with NC. This sample was therefore designated as Mo_2_C@NC, and was exclusively subjected to subsequent detailed analyses.

The cross‐linked coating structure was confirmed by scanning electron microscopy (SEM). As shown in Figures S10a,b and 2d, the cross‐sectional SEM images clearly reveal the interconnected network‐like skeleton with prominent macropores formed during the phase separation process. In contrast, both the top and bottom surfaces of the membrane exhibit porous morphologies (Figure S10c,d), further confirming the formation of hierarchical porous structure. The Mo_2_C@NC was coated with the C/S composite and directly used as the cathode without Al foil (Figure S10e,f).

Transmission electron microscopy of the microreactor was performed to further give evidence of the coating structure (Figure 2e) with an amorphous carbon matrix encapsulating the Mo_2_C core (Figure 2f,g). The exposed lattice fringes correspond to the (311) plane of Mo_2_C, which is indexed to PDF#15‐0457 (Figure 2h). Elemental mapping analysis (Figure 2i–m) confirms the homogeneous distribution of Mo, C, and N throughout the material, Moreover, elemental mapping indicates that carbon is more widely distributed than Mo_2_C, further confirming the core‐shell structure between the N‐doped carbon layer and the Mo_2_C core. To further confirm the successful synthesis of the material, XRD measurements were conducted (Figure 2n). The obtained Mo_2_C@NC composite shows diffraction peaks consistent with Mo_2_C (PDF#15‐0457); a broad peak observed in the 2*θ* range of 20–30° further confirms the presence of amorphous carbon.

To further elucidate the characteristics of the obtained material, the physical properties of Mo_2_C@NC were systematically analyzed. Raman spectroscopy (Figure 2o) shows two prominent peaks, D and G bands, corresponding to the disorder‐induced and graphitic carbon structures, respectively. The higher *I*_D_/*I*_G_ ratio of the MoO_3_ precursor indicates a larger degree of structural defects, which may facilitate improved catalytic activity. Through the nitrogen adsorption–desorption measurements (Figure S11), it can be obtained that the specific surface area of the material is 23.51 m^2^/g and the pore volume is 0.12 cm^3^/g. Thermogravimetric analysis was further performed in air with a heating rate of 10°C min^−1^ to quantify the Mo_2_C content (Figure 2p). The sample exhibited an initial weight gain followed by a sharp decline, stabilizing above ∼500°C. Based on the oxidation reaction (Mo_2_C + 4O_2_ → 2MoO_3_ + CO_2_), the mass conversion factor (*k*) from Mo_2_C to the solid oxidation product MoO_3_ is given by

$$w_{{\text{Mo}}_{2}C}=\frac{m_{\text{res}}}{m_{0}}\frac{M\left({\text{Mo}}_{2}C\right)}{2M\left(\text{Mo}O_{3}\right)}=0.512\times \frac{203.91}{2\times 143.95}=0.3626,$$

where ​m_res_ and​ *m*_0_ represent the initial sample mass and the stabilized residual mass after complete oxidation, respectively, the Mo_2_C content in the composite was calculated to be approximately 36.26%.^[28]^

Mo_2_C@NC, NC, and carbon‐coated aluminum foil (denoted as Al/C) were used as cathode materials in lithium–sulfur coin cells. After resting for 12 h, electrochemical impedance spectroscopy (EIS) was conducted directly (Figure 3a). The corresponding equivalent circuit model and calculated result are given in Figure S12 and Table S1, where R1 is the ohmic impedance, R2 is the interface impedance and R3 is the charge transfer impedance. Among all electrodes, the Mo_2_C@NC‐based cell exhibited the lowest impedance, indicating enhanced interfacial charge transfer and improved overall conductivity, which are beneficial for battery performance. Moreover, the calculated Li^+^ diffusion coefficients (Figure 3b) calculated based on the equation^[29]^ $D_{{\text{Li}}^{+}}=\frac{R^{2}T^{2}}{0.5A^{2}n^{4}F^{4}C^{4}\sigma^{2}}$ and $Z^{\prime }=R_{e}+R_{ct}+\sigma \omega^{-0.5}$ further confirm the fastest ion transport and electron migration within the Mo_2_C@NC electrode. These results demonstrate that the incorporation of Mo_2_C effectively optimizes the interfacial electronic structure of NC, thereby promoting the rapid redox kinetics and enhancing the overall electrochemical performance. To further probe the redox kinetics of redox conversion, symmetric cells based on the Mo_2_C@NC membrane were assembled and evaluated (Figure 3c). Compared with NC, the Mo_2_C@NC symmetric cell displays markedly higher current for all redox pairs (10.3 and 17.0 A g^−1^), while peaks for NC only display 2.5 and 4.6 A g^−1^ at each. This gives evidence of signifying faster LiPSs redox conversion kinetics facilitated by the synergistic electronic interaction between Mo_2_C and NC.^[30]^

**FIGURE 3:**
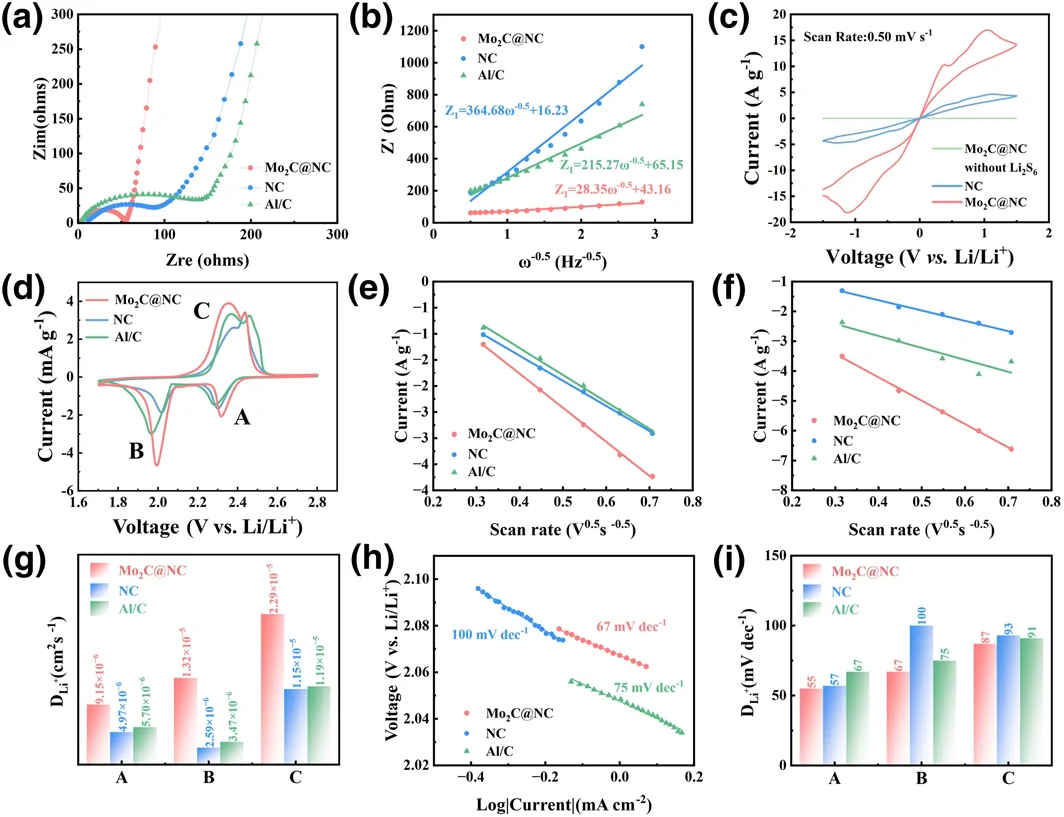
Electrochemical experiment of Mo_2_C@NC, nitrogen‐doped carbon (NC), and aluminum foil. (a) Electrochemical impedance spectroscopy and (b) corresponding Li^+^ diffusion rate. (c) Symmetric cyclic voltammetry (CV) tests of three samples. (d) CV tests of Li‐S cells assembled with Mo_2_C@NC, NC and Al/C cathode; peak currents of CV at (e) peak A and (f) peak B. (g) Calculated lithium‐ion diffusion coefficients. (h) Tafel plots corresponding to peaks A. (i) Comparation of Tafel slopes at peak A, B, and C.

To gain deeper insight into the catalytic modulation of Mo_2_C to the electrochemical behavior of NC, cyclic voltammetry (CV) was carried out for Li‐S cells assembled with Mo_2_C@NC, NC and Al/C cathode in the voltage range of 1.7–2.8 V. As shown in Figure 3d, at a scan rate of 0.2 mV s^−1^, all cells display two cathodic peaks in the discharge process, located at 2.2–2.4 V (peak A) and 1.9–2.1 V (peak B), corresponding to the reduction of elemental sulfur (S_8_) to soluble polysulfides (Li_2_S_n_, 4 ≤ *n* ≤ 8), and the subsequent conversion of soluble polysulfides to insoluble Li_2_S_2_/Li_2_S, respectively. The broad anodic peak between 2.4 and 2.6 V (peak C) corresponds to the reverse oxidation process.^[31]^ Notably, the Mo_2_C@NC electrode exhibits the most pronounced redox peaks among the samples, with its anodic peak shifted more negatively and cathodic peaks more positively, indicating reduced polarization and accelerated redox kinetics due to the synergistic effect between Mo_2_C and NC. This behavior can be well correlated with the electronic structure modulation revealed by the DFT calculations. As discussed above, the coupling between Mo_2_C and pyridinic N shifts the p‐band center of pyridinic N closer to the Fermi level, which enhances the electron exchange capability of the active sites toward LiPS intermediates. As a result, the Mo_2_C@NC electrode exhibits accelerated sulfur redox reactions and improved charge‐transfer kinetics, which are reflected by the reduced polarization and enhanced peak currents observed in the CV profiles. To further quantify the lithium‐ion diffusion kinetics, CV measurements were conducted at scan rates ranging from 0.1 to 0.5 mV s^−1^. Peak currents at A, B, and C were extracted to calculate the diffusion coefficients using the Randles‐Sevcik equation (Figure 3e,f and Figure S14).

$$i_{p}=\left(2.69\times 10^{5}\right)n^{\frac{3}{2}}AD^{\frac{1}{2}}Cv^{\frac{1}{2}},$$

where $i_{p}$ is the peak current (A), n is the number of electrons transferred, A is the electrode area (cm^2^), D is the diffusion coefficient (cm^2^ s^−1^), C is the concentration of the electroactive species (mol cm^−3^), and v is the scan rate (V s^−1^). This equation is valid at 298 K.

The calculated lithium‐ion diffusion coefficients for Mo_2_C@NC were 9.15 × 10^−6^, 1.32 × 10^−5^, and 2.29 × 10^−6^ cm^2^ s^−1^ for peaks A, B, and C, respectively, while those for NC were 4.97 × 10^−6^, 2.59 × 10^−6^, and 1.15 × 10^−5^ cm^2^ s^−1^, respectively (Figure 3g). These results confirm the superior ionic transport in Mo_2_C@NC and suggest that the presence of Mo_2_C effectively alleviates concentration polarization and promotes fast electrochemical kinetics.

Tafel plots corresponding to peak A, B, and C were extracted from the regions just before the slope transition near the three characteristic peaks at the scan rate of 0.2 mV s^−1^ (Figures 3h and S15). As shown in Figure 3i, the Mo_2_C@NC sample exhibited the smallest Tafel slopes among all groups, indicating the highest catalytic reaction rate and the most favorable redox kinetics.

On this basis, the Gibbs free energy diagrams of the rate‐determining step during the sulfur reduction process (from S_8_, Li_2_S_4_, Li_2_S_4_ to Li_2_S) were calculated on the surfaces of pure pyridinic nitrogen, Mo_2_C, and pyridinic N coupled with Mo_2_C, the corresponding configurations of Li_2_S adsorption on surface is also given (Figures 4a and S16). Among these, Mo_2_C@NC exhibits the lowest energy barrier, confirming that the incorporation of Mo_2_C significantly reduces the conversion energy barrier of NC in the rate‐determining step, thereby facilitating the overall redox of the battery. X‐ray photoelectron spectroscopy (XPS) analysis was conducted on N atom in NC before and after binding with Mo_2_C, the arithmetic mean values of all N peaks were calculated (indicated by the red dotted line), showing an overall negative shift (Figure 4b). This indicates an electron transfer from Mo_2_C to nitrogen atoms leading to an increased electron cloud density, which is consistent with the simulation results. Moreover, XPS fitting analysis of N (Figure 4c) of the Mo_2_C@NC membrane before and after Li_2_S_6_ adsorption clearly shows that all nitrogen peaks undergo a positive shift. This is attributed to the adsorption‐induced decrease in nitrogen electron cloud density. As for Mo atoms, the Mo^2+^ peak shifts lower, suggesting an increase in electron density at the Mo center because of its electron contribution to NC (Figure S17). The adsorption capability of the material was further visualized through Li_2_S_6_ adsorption experiment. 10 mg of Mo_2_C@NC and NC membrane debris were dispersed in Li_2_S_6_ solution under the same concentration (Figure 4d). After 24 h of standing, the solution containing Mo_2_C@NC debris turned nearly transparent. UV‐vis spectra further confirm this observation where the characteristic peak at 450 nm almost disappeared for the Mo_2_C@NC sample, whereas the peak remains in the control group, thereby verifying its superior adsorption capacity. The enhanced Li_2_S_6_ adsorption capability of Mo_2_C@NC can be correlated with the electronic structure modulation revealed by DFT calculations. The coupling between Mo_2_C and pyridinic N shifts the p‐band center closer to the Fermi level, strengthening the electronic interaction between the active sites and LiPS species, thereby improving Li_2_S_6_ immobilization. In addition, the Li_2_S deposition performance, which represents the rate‐determining step of Li–S batteries, was investigated (Figure 4e). A distinct deposition peak corresponding to Li_2_S was observed for Mo_2_C@NC at 330 s with a deposition capacity of 390 mAh g^−1^, while NC exhibited only a weak deposition signal after 1000 s. This highlights the superior catalytic capability of Mo_2_C@NC toward the control steps of the reaction (Figure S18).

**FIGURE 4:**
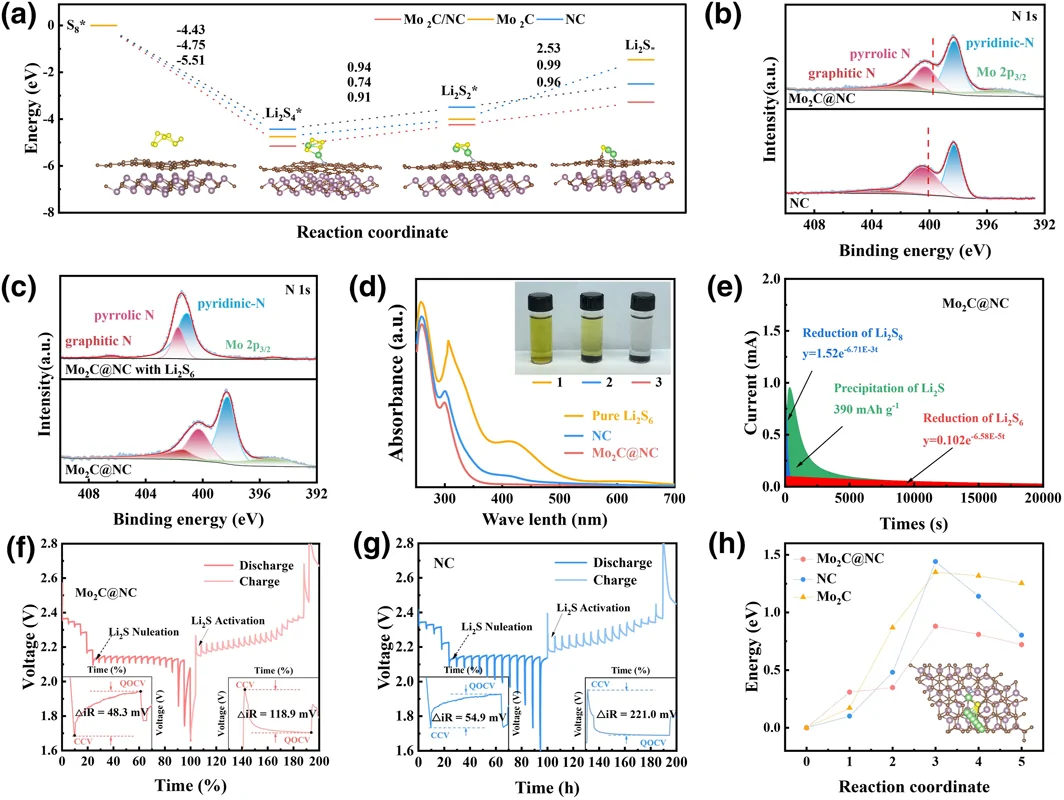
(a) Gibbs free energy diagrams of the rate‐determining step during the sulfur reduction process (from S_8_, Li_2_S_4_, Li_2_S_4_ to Li_2_S). (b) X‐ray photoelectron spectroscopy (XPS) analysis conducted on N in nitrogen‐doped carbon (NC) before and after binding with Mo_2_C. (c) XPS fitting analysis of N for the Mo_2_C@NC membrane before and after Li_2_S_6_ adsorption. (d) Li_2_S_6_ adsorption experiment for Mo_2_C@NC and NC membrane debris and corresponding UV–vis spectra of the solution. (e) The Li_2_S deposition behavior of Mo_2_C@NC. (f, g) Galvanostatic intermittent titration technique analysis for cell assembled with Mo_2_C@NC and NC. (h) The dissociation process of Li_2_S.

Furthermore, galvanostatic intermittent titration technique analysis was conducted to monitor the internal resistance with respect to normalized discharge/charge time (Figure 4f,g). During Li_2_S nucleation, the internal resistance (ΔiR) of Mo_2_C@NC was only 48.3 mV, compared to 54.9 mV for NC. During Li_2_S_4_ dissolution, Mo_2_C@NC also exhibited the lowest ΔiR of 118.9 mV, significantly lower than that of NC (221.0 mV). These results confirm that Mo_2_C induces substantial electronic redistribution within NC, thereby promoting the conversion of sulfur species. The dissociation process of Li_2_S is also examined in Figures 4h and S19, and the configurations of Li_2_S adsorption on the surface of PyNC and Mo_2_C are also given. NC coupled with Mo_2_C possesses the lowest dissociation energy barrier, which further reveals that the Mo_2_C@NC microreactor accelerates the reaction kinetics during the charging process of Li‐S batteries.^[32]^

To further elucidate the charge‐transfer characteristics of the Mo_2_C@NC electrode, in situ measurements including in situ EIS, XRD, and Raman spectroscopy were performed. In situ EIS tests were performed and shown in Figure 5a,b, with the fitting parameters summarized in Table S2 and S3. During the discharge process, the *R*_ct_ evolution of both Mo_2_C@NC and NC follows a typical trend of first increasing followed by gradual decreasing, reflecting the solid‐liquid‐solid conversion pathway of LiPSs. Notably, within the kinetically sluggish voltage region (2.05–1.7 V), the *R*_ct_ of Mo_2_C@NC remains nearly unchanged, whereas that of NC rises dramatically, exceeding its initial value by more than an order of magnitude. This behavior highlights the ability of Mo_2_C@NC to alleviate resistance growth at the rate‐determining step. In addition, the *R*_ct_ values of Mo_2_C@NC are consistently lower than those of NC throughout the entire process, further contributing to the enhanced redox kinetics (Figure 5c).

**FIGURE 5:**
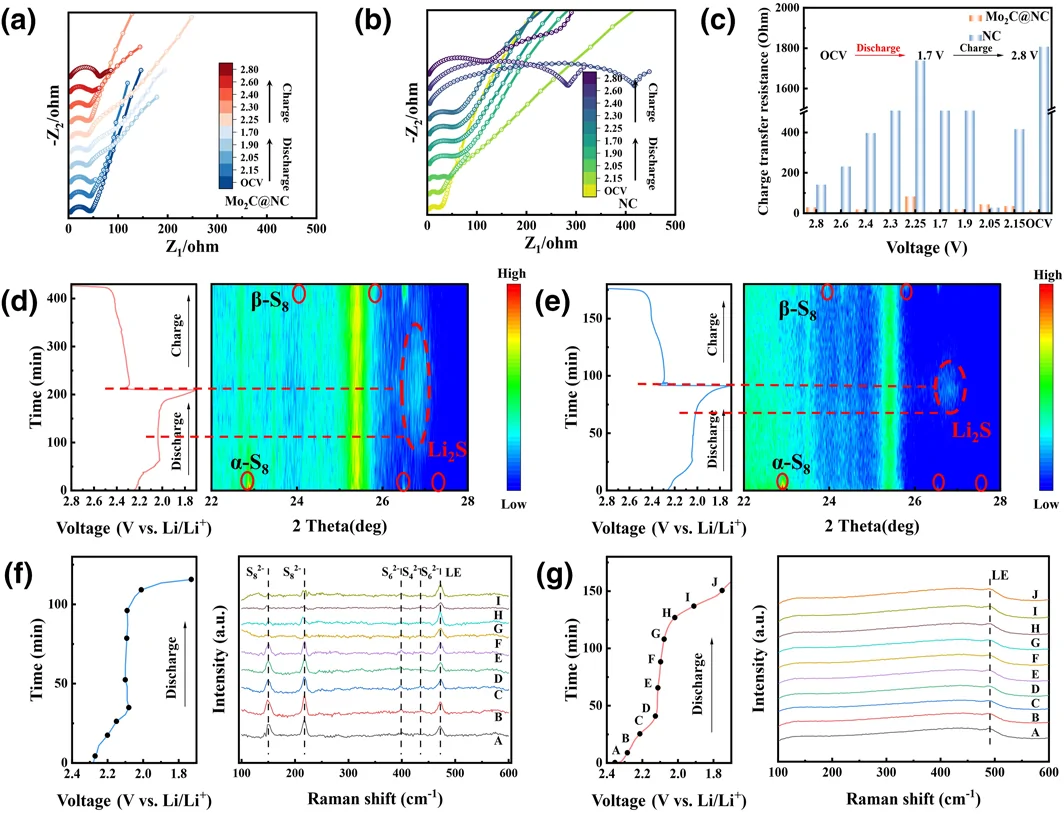
In situ tests of Mo_2_C@NC and nitrogen‐doped carbon (NC) electrode. (a, b) In situ electrochemical impedance spectroscopy analysis. (c) The *R*_ct_ values of the batteries. (d, e) In situ X‐ray diffraction analysis. (f, g) In situ Raman analysis.

In situ XRD analysis was further carried out to monitor the phase evolution and Li_2_S formation process of Mo_2_C@NC cathode during the charge‐discharge process^[33]^ (Figure 5d,e). For the battery without Mo_2_C, the high kinetic barrier led to an incomplete reduction of α‐S_8_, leaving most of the sulfur trapped as intermediate LiPSs and only a limited fraction converted into Li_2_S. In sharp contrast, the Mo_2_C@NC electrode displayed nearly full conversion of sulfur species into Li_2_S as evidenced by the strong Li_2_S diffraction signals during discharge, which subsequently reverted to crystalline sulfur upon charging. These observations confirm that Mo_2_C@NC not only suppresses polysulfide accumulation but accelerates their catalytic conversion. In situ Raman spectroscopy was also employed to track polysulfide evolution during discharge^[34]^ (Figure 5f,g). For the NC electrode, characteristic S_8_ signals at 150 and 219 cm^−1^ persisted during the first plateau (∼2.3 V). As the potential reached the second plateau (∼2.05 V), the intensities of these peaks diminished, while new features around 400 and 450 cm^−1^ appeared, corresponding to Li_2_S_4_ and Li_2_S_6_ species. A broad band near 500 cm^−1^, arising from the electrolyte, was also detected. In sharp contrast, the Mo_2_C@NC electrode exhibited much weaker peaks during discharge, suggesting more rapid polysulfide conversion and effective suppression of soluble intermediates.

To evaluate the long cycle life of electrochemical performance of the Mo_2_C@NC electrode, Li‐S cells were assembled using Mo_2_C@NC, NC, and carbon‐coated aluminum foil (Al/C) as cathodes, respectively. After three cycles of activation, rate capability tests were conducted at current densities of 0.2, 0.5, 1.0, 2.0, and 4.0 C (Figure 6a). The Mo_2_C@NC‐based cell exhibited excellent rate performance with discharge capacities of 1235.5, 1043.0, 963.7, 882.7, and 775.3 mAh g^−1^ at each, significantly better than the NC‐based electrode (992.0, 802.0, 706.2, 604.9, and 465.6 mAh g^−1^). Upon returning to 0.2 C, the capacity of the Mo_2_C@NC cell recovered to 1093.2 mAh g^−1^, maintaining 88.5% of its initial value, whereas the NC electrode only recovered to 798.2 mAh g^−1^ with retention of 80.0%, indicating better reversibility in Mo_2_C@NC electrode. The corresponding charge–discharge profiles of different current rates are shown in Figure S20. Notably, at 0.2 C (Figure 6b), the Mo_2_C@NC electrode displays the longest discharge plateau, especially in the low‐voltage region (plateau II). The capacity ratio between the low‐voltage plateau (*Q*_L_) and high‐voltage plateau (*Q*_H_) is significantly higher than that of the control groups (Figure 6c), confirming the enhanced catalytic conversion of polysulfides. Furthermore, the voltage polarization between charge and discharge plateaus for Mo_2_C@NC is only 0.1643 V, significantly lower than that of NC and Al/C, reflecting its superior redox kinetics and reduced internal resistance.

**FIGURE 6:**
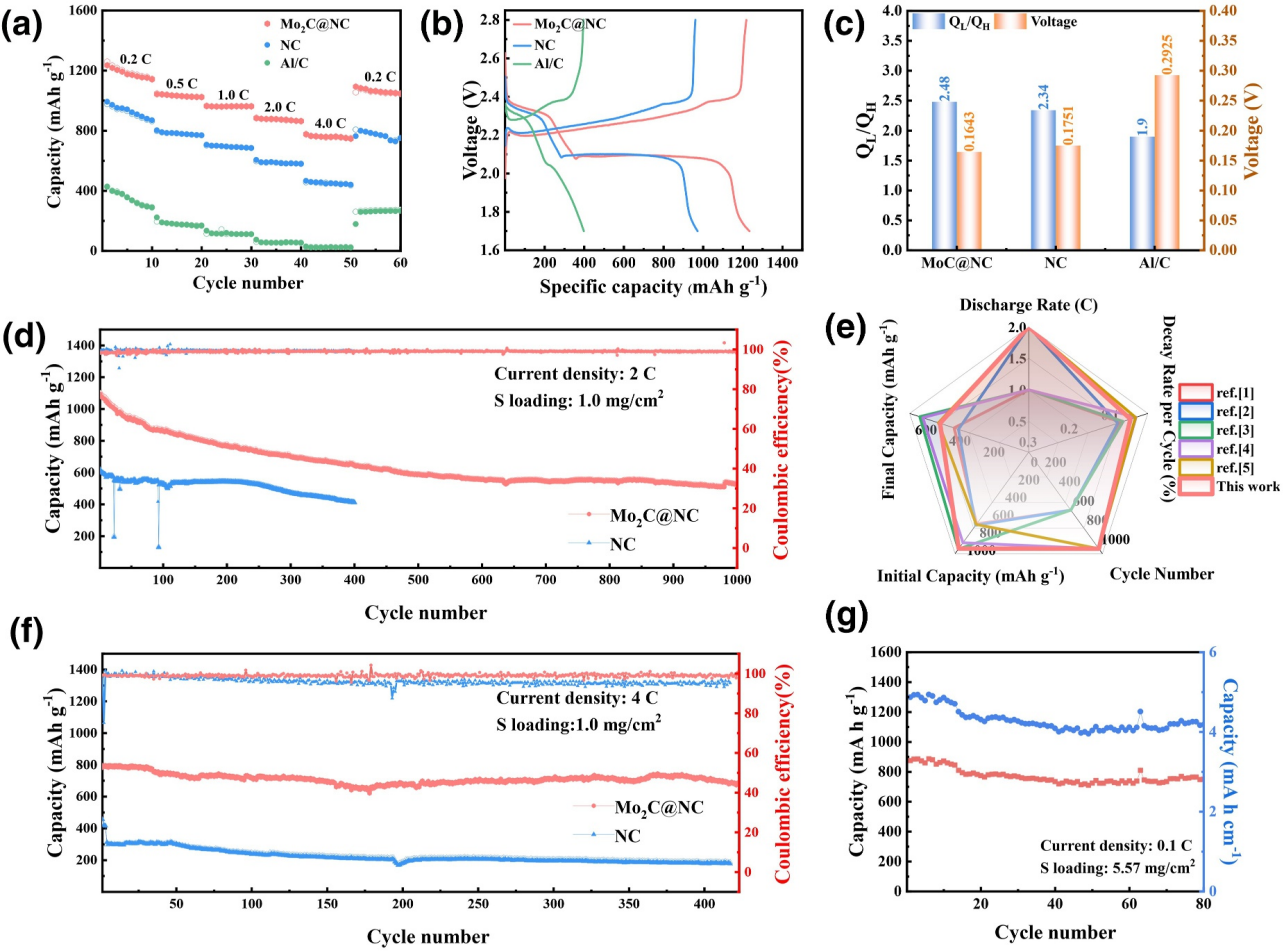
(a) Rate performance of three cathodes, and (b) the corresponding charge/discharge profiles at 0.2 C. (c) The capacity ratio between the low‐voltage plateau (*Q*_L_) and high‐voltage plateau (*Q*_H_) and the voltage polarization between charge and discharge plateaus. (d) Cycling performance of Mo_2_C@NC cathodes at 2.0 C. (e) Comparation with other Mo_2_C‐based modification strategies. (f) Cycling performance of Mo_2_C@NC cathodes at 4.0 C. (g) Cycling performance of Mo_2_C@NC cathodes under high sulfur loading (5.57 mg cm^−2^).

Benefiting from these advantages, the Mo_2_C@NC electrode achieved an initial capacity of 1093.6 mAh g^−1^ at 2.0 C and maintained stable cycling over 1000 cycles with an ultralow capacity decay rate of only 0.0523% per cycle (Figure 6d), which significantly outperforms the NC‐based counterpart. Compared with other Mo_2_C‐based modification strategies[^35^, ^36^, ^37^, ^38^, ^39^] (Figure 6e), the Mo_2_C@NC electrode demonstrates a much‐improved long‐term cycling stability, confirming the stabilizing sulfur redox reactions. Even under high‐rate conditions at 4.0 C (Figure 6f), the Mo_2_C@NC cell delivers an initial capacity of 790.6 mAh g^−1^ remained stable cycling for over 420 cycles, and an exceptionally low fading rate of 0.027% per cycle. Since the effective donation of Mo_2_C to NC, the coulombic efficiency of Mo_2_C has been relatively higher, proving its better cycling stability. Furthermore, under a high sulfur loading of 5.57 mg cm^−2^, the cell retained stable cycling for 80 cycles with a high areal capacity of 4.87 mAh cm^−2^, demonstrating excellent practical application potential (Figure 6g). The excellent cycling stability of Mo_2_C@NC can also be associated with the electronic coupling between Mo_2_C and pyridinic N. The strengthened LiPS adsorption and accelerated conversion effectively suppress the shuttle effect and maintain stable sulfur redox reactions during long‐term cycling.

## 3 CONCLUSION

In summary, this work presents a microreactor‐based design strategy guided by theoretical insights for advanced Li–S battery cathodes. DFT calculations revealed that coupling NC with Mo_2_C induces pronounced electron redistribution, with pyridinic N showing the strongest electronic interaction and the most favorable p‐band alignment for LiPSs adsorption and conversion. Guided by these insights, a phase‐inversion strategy was employed to fabricate a MoO_3_@PAN precursor. Subsequent carbonization produced a 3D Mo_2_C@NC microreactor consisting of a pyridinic‐N‐rich NC shell strongly coupled with Mo_2_C core. This architecture achieves zero‐distance integration of conductivity and catalytic active sites. As a result, the Mo_2_C@NC cathode exhibits outstanding electrochemical performance, including a long cycling life of 1000 cycles with a low decay rate of 0.0523% per cycle and excellent rate capability, maintaining 790.6 mAh g^−1^ at 4.0 C for 400 cycles. These results highlight that integrating the microreactor architecture with electronic structure modulation provides an effective strategy for accelerating polysulfide conversion and developing robust, high‐performance Li–S battery cathodes.

## CONFLICT OF INTEREST STATEMENT

The authors declare no conflicts of interest.

## ETHICS STATEMENT

This article does not contain any studies with human participants or animals performed by any of the authors.

## Supporting information

Supporting Information S1

## Data Availability

The data that support the findings of this study are available from the corresponding author upon reasonable request.
