# Supplementary material for: Phase‐inversion constructed Mo2C@NC microreactor with optimized pyridinic N p‐band center for high‐performance Li–S batteries
Source: Smart Mol. 2026 May 6;4(3):e70051. doi: 10.1002/smo2.70051 (PMC13399107; doi:10.1002/smo2.70051)
Supplement: Supplementary file 1 [file SMO2-4-e70051-s001.docx]

**Supporting information**

**Phase-Inversion Constructed Mo_2_C@NC Microreactor with Optimized Pyridinic N p-Band Center for High-Performance Li–S Batteries**

1. **Chemicals**

Ammonium molybdate (H_24_Mo_7_N_6_O_24_·4H_2_O, Ar)，Polyacrylonitrile (PAN, MW = 150000, > 99%) and N,N-dimethylformamide (DMF, > 99.9%) was purchased from Aladdin. Nitric acid (HNO_3_, Ar) was purchased from Xilong Scientific. Sulfur was purchased from Tianjin Damao Chemical Reagent Factory. Polyvinylidene fluoride (PVDF-5130) and N-methyl pyrrolidone (NMP, AR) was purchased from Shenzhen Kelude Company. All the commercially available chemicals were used without further purification.

2. **Synthesis of MoO_3_ nanowire**

Typically, 2.0 g H_24_Mo_7_N_6_O_24_·4H_2_O, 9.5 mL HNO_3_ and 47.5 mL H_2_O was mixed and stirred continuously. After stirring for 30 min, the solution was transferred into a 100 mL polytetrafluoroethylene-lined reaction vessel and subjected to a hydrothermal reaction at 200°C for 20 hours to yield MnO_3_ nanowire. The resulting white product was collected by filtration and rinsed with deionized water and ethanol for several times, followed by drying at 60 °C.

3. **Preparation of carbon-coated porous nanowire membranes:**

The membrane was prepared based on phase inversion method. Basically, a polymer casting solution containing nanofillers is immersed in a nonsolvent bath, triggering rapid phase separation. During this process, liquid–liquid demixing produces polymer-rich and polymer-lean phases. The polymer preferentially deposits around the nanofillers, forming a dense and defect-free encapsulation layer. This in-situ encapsulation avoids interfacial defects of polymers and enables the formation of 3D nanonetworks with 1D inorganic nanofillers as cores and cross-linked polymer shell.

In this work, the as-prepared nanowire was mixed with polyacrylonitrile (PAN) and N, N-dimethylformamide (DMF) in a 1:1:10 weight ratio and stirred at room temperature. The resulting mixture was cast onto a glass plate using a doctor blade at room temperature (25℃), while maintaining the environmental humidity below 80%. The casted glass plate was then rapidly transferred into 2 L deionized water to induce phase inversion. After standing for 24 h, a liquid film with a cross-linked MoO₃@PAN network structure was obtained After drying at 60℃ overnight, the white film transferred to muffle oven and pretreated in air at 250℃ for 2h. After naturally cooled to room temperature, the film was cut into 16mm pieces. The wafer was later placed between two corundum plates and put into a tube furnace. The temperature of the tube was raised at a rate of 5°C per minute to 700°C under an argon atmosphere, maintained for 3 hours, and then naturally cooled to room temperature. The resultant product was named Mo_2_C@NC. NC was prepared by the same method except for adding MoO_3_ nanobelt.

4. **Cell Assembly and Test**
1) **Synthesis of cathode**

The cathode of the battery is fabricated by coating C/S slurry directly on to Mo_2_C@NC membrane. C/S powder is prepared by grinding carbon black and sulfur for 30 min from which the proportion of sulfur is 62.5%. Later, the powder is transferred to 20 mL polytetrafluoroethylene-lined reaction vessel and treated at 155℃ for 12 h in Ar atmosphere. The uniform C/S slurry is prepared by combining 90 mg of C/S powder with 10 mg of polyvinylidene fluoride (PVDF-5130) as the binder, and adding 700 μL of N-methyl pyrrolidone as the solvent. After stirring for 4 h, the resulting mixture is applied onto Mo_2_C@NC membrane using a pipette tip to achieve a controlled sulfur loading in the range of 1-5 mg cm^-2^. The cathode is finally obtained by drying the coated disc in a vacuum oven at 60 °C overnight.

2) **Electrochemical Measurements Characterization**

The CR2025 coin cells are assembled with C/S coated Mo_2_C@NC membrane as the cathode, lithium plate (64 mg, ~2 cm^2^) as anode and Celgard 2025 (PP) as separator. Al the cells are assembled in an argon-filled glove box with the H_2_O and Air level of less than 0.1 ppm. The electrolyte for the battery is a mixture of LiTFSI (1 M) with LiNO_3_ (2 wt.%), which is dissolved in 1,2-dimethoxyethane (DME) and 1,3-dioxolane (DOL) (1:1, v/v). The long cycle performance of the assembled batteries is tested at 30 ℃ by using LAND CT2001A multichannel battery test system with a voltage window of 1.7-2.8 V. Cyclic voltammetry (CV) profiles are performed at scan rates of 0.1, 0.2, 0.3, 0.4, and 0.5 mV s^-1^. Electrochemical impedance spectroscopy (EIS) profiles ranged from 100 kHz to 0.01 Hz.

3) **Assembly of Symmetric Cells and Kinetic Study**

Symmetric cyclic voltammetry (CV) tests were conducted using CR2025 coin cells with Mo_2_C@NC as both the working and counter electrodes, placed on either side of a polypropylene (PP) separator. A 0.2 M Li_2_S_6_ solution was prepared by dissolving lithium sulfide (Li_2_S) and sulfur in a molar ratio of 1:5 into 1.0 M LiTFSI in a mixed solvent of DME/DOL (1:1 by volume). For each cell, 50 µL of the 0.2 M Li_2_S_6_ solution and 10 µL of electrolyte were used. The CV tests were carried out at a scan rate of 0.5 mV s⁻¹ within a voltage window of -1.5 to 1.5 V.

4) **Li_2_S nucleation and Decomposition Tests**

The nucleation behavior of Li_2_S was investigated using CR2025 coin cells assembled with Mo_2_C@NC membranes as cathodes and lithium metal as the anode. For each cell, 40 μL of Li_2_S₈ catholyte—prepared by dissolving Li_2_S and sulfur in a 1:7 molar ratio in 1.0 M LiTFSI (DME/DOL, 1:1 by volume)—was added to the cathode side. During the nucleation test, the cells were discharged to 2.05 V at a constant current of 0.112 mA, followed by amperometric i–t measurements at an initial voltage of 2.05 V until the current dropped below 10 μA. All electrochemical measurements were performed using a Princeton Applied Research electrochemical workstation. Control cells using NC were assembled following the same procedure.

5. **Characterization details:**

X-ray diffraction (XRD) patterns of the samples were recorded using a Bruker D8 ADVANCE X-ray diffractometer with Cu Ka radiation. The morphology and cross section of the membrane were observed by scanning electron microscopy using a JSM-7610F Plus emission scanning electron microscope coupled with an energy-dispersive X-ray (EDX) spectrometer. High-resolution transmission electron microscopy (HRTEM, JEM-2100F) and scanning transmission electron microscopy (STEM) with Super EDS mapping being carried out at 300 kV using a FEI/Thermo scientific Themis Z (60-300 kV) was used for high magnification observation.The X-ray photoelectron spectroscopy (XPS, ESCALAB XI+) technique is used to determine the chemical states of the elements and identify the nature of interactions present between the constituents. The Raman spectroscopy (InVia Qontor) was collected with 532 nm laser excitation under ambient conditions.

6. Calculations details:

The density functional theory (DFT) calculations were performed using the open-source CP2K software package with the generalized gradient approximation Perdew-Burke-Ernzerhof (GGA-PBE) functional to describe the exchange-correlation effects. Goedecker-Teter-Hutter (GTH) pseudopotentials were employed to represent the ionic cores, while valence electrons were described using a mixed Gaussian and plane-wave (GPW) basis set. A plane-wave kinetic energy cutoff of 500 eV (approximately 37 Ry, adjusted to CP2K’s Rydberg units) was used for the auxiliary plane-wave grid to ensure accurate representation of the electron density. The electronic energy convergence criterion was set to a total energy change smaller than 10^-4^ eV.

Geometry optimizations were conducted with a residual force threshold of 10^-2^ eV/Å (approximately 0.0194 Ha/Bohr in CP2K units). The Climbing Image-Nudged Elastic Band (CI-NEB) method was implemented in CP2K to calculate the migration barriers of Li ions within the structures. The surface was modeled using a symmetric periodic slab, with a 15 Å vacuum layer inserted along the z-direction to minimize interactions between the slab and its periodic images.

The *d* or p band center is defined by the following equation:

$\text{ε}_{\text{d, p}}\text{=}\frac{\int_{\text{-∞}}^{\text{∞}} \text{n}_{\text{d, p}}\left( \text{ε} \right)\text{εdε}}{\int_{\text{-∞}}^{\text{∞}} \text{n}_{\text{d}},p\left( \text{ε} \right)\text{dε}}$

where n_d,p_(ε)*n_d_*_,_*_p_*(*ε*) represents the density of states for the d or p orbitals, obtained from the projected density of states (PDOS) analysis in CP2K.

The binding energy of lithium polysulfides on the surfaces is computed as:

$\text{E}_{\text{b}}\text{=}\text{E}_{\text{total}}\text{-}\text{E}_{\text{sub}}\text{-}\text{E}_{\text{LiPSs}}$

Where *E_sub_* and *E_S8/LiPSs_* are the energy of the bare surfaces and LiPSs, respectively, and *E_total_* was the total energy of the configurations of different surfaces surface.

The corresponding reaction energies for the two steps were calculated from the equation (1, 2), respectively:

$E_{\text{L}\text{i}_{2}S_{2}+\text{substrate}}-E_{\text{L}\text{i}_{2}S_{4}+\text{substrate}}+1/4E_{S_{8}}$ （3）

$E_{\text{L}\text{i}_{2}S+\text{substrate}}+\frac{1}{8}E_{S_{8}}-E_{\text{L}\text{i}_{2}S_{2}+\text{substrate}}$（4）

where E_Li_ is the energy of lithium metal per atom and E_S8_ is the gas-phase energy of S_8_.

**Figure S1** Configurations of graphitic N, pyrrolic N, and pyridinic N

**Figure S2** Configurations of GraNC, PrNC, and PrNC after integrated with Mo_2_C, denoted as GraNC/Mo_2_C, PrNC/Mo_2_C, and PrNC/Mo_2_C

**Figure S3** Configurations of GraNC/Mo_2_C and PrNC/Mo_2_C after adsorption of Li_2_S_4_ and the corresponding binding energy.

**Figure S4** Density of states (DOS) of GraNC, PrNC, and PyNC before and after coupling with Mo_2_C

**Figure S5** (a) XRD pattern of MoO_3_@PAN, and (b) SEM image of MoO_3_@PAN

**Figure S6** Synthesis process of Mo_2_C@NC

**Figure S7** XRD pattern of MoO_3_

**Figure S8** XRD pattern of M3, M4, M5, M6, M7, and M8

**Figure S9** Batteries assembled with M3, M4, M5, M6, M7, and M8 cycled at 1.0 C

**Figure S10** SEM image of Mo_2_C@NC (a-b) cross-sectional, (c) top, and (d) bottom surfaces; (e) cross-sectional and (f) top surface image of Mo2C@NC covered with C/S composite

**Figure S11** Nitrogen adsorption–desorption measurements of Mo_2_C@NC and NC

Where R1 is ohmic impedance, R2 is the interface impedance and R3 is the charge transfer impedance.

**Figure S12** Equivalent circuit model of the assembled battery

**Figure S13** CV measurements conducted at scan rates ranging from 0.1 to 0.5 mV s⁻¹

**Figure S14** CV Peak currents at peak C

**Figure S15** Tafel plots corresponding to peak B and C

**Figure S16** Configurations of Li_2_S adsorption on surface of PyNC and Mo_2_C

**Figure S17** The Li_2_S deposition performance of NC

**Figure S18** Configurations of Li_2_S dissociation process for PyNC and Mo_2_C

**Figure S19** (a) XPS fitting analysis of Mo 3d for the Mo_2_C@NC membrane before and after Li_2_S_6_ adsorption and (b) S 2p for the Mo_2_C@NC membrane after Li_2_S_6_ adsorption

**Figure S20** Charge–discharge profiles of different current rates

**Table S1.** Specific impedance values of R1, R2 and R3 of Mo2C@NC battery during in-situ EIS

|  | R1 | R2 | R3 |
| --- | --- | --- | --- |
| Mo2C@NC | 1.99 | 29.39 | 25.21 |
| NC | 3.826 | 429.3 | 47.63 |
| Al/C | 2.369 | 93.9 | 61.38 |

**Table S2.** Specific impedance values of R1, R2 and R3 of Mo_2_C@NC battery during in-situ EIS

| Voltage (V) | R1 | R2 | R3 |
| --- | --- | --- | --- |
| 2.8 | 2.901 | 28.8 | 29.14 |
| 2.6 | 2.203 | 34.97 | 5.807 |
| 2.4 | 4.363 | 20.27 | 18.73 |
| 2.3 | 4.649 | 29.84 | 7.472 |
| 2.25 | 3.632 | 31.5 | 83.44 |
| 1.7 | 4.156 | 28.9 | 6.939 |
| 1.9 | 4.662 | 8.266 | 19.87 |
| 2.05 | 3.972 | 36.49 | 43.84 |
| 2.15 | 3.502 | 36.92 | 35.42 |
| OCV | 2.522 | 25.43 | 14.03 |

**Table S3.** Specific impedance values of R1, R2 and R3 of NC battery during in-situ EIS

| Voltage (V) | R1 | R2 | R3 |
| --- | --- | --- | --- |
| 2.8 | 2.118 | 105 | 141.7 |
| 2.6 | 1.208 | 196.1 | 231.3 |
| 2.4 | 1.145 | 213.2 | 397.5 |
| 2.3 | 2.319 | 60.01 | 890.3 |
| 2.25 | 2.344 | 105.8 | 1738 |
| 1.7 | 2.188 | 94.29 | 1413 |
| 1.9 | 2.177 | 92.27 | 1387 |
| 2.05 | 2.642 | 21.32 | 27.66 |
| 2.15 | 2.384 | 26.16 | 416.6 |
| OCV | 2.033 | 32.87 | 1807 |
